# Applicability of the Clavien-Dindo classification to emergency surgical procedures: a retrospective cohort study on 444 consecutive patients

**DOI:** 10.1186/1754-9493-8-31

**Published:** 2014-07-26

**Authors:** Panu J Mentula, Ari K Leppäniemi

**Affiliations:** 1 Department of Abdominal Surgery, Helsinki University Central Hospital, P.O. Box 340, 00029 HUS Helsinki, Finland

**Keywords:** Emergency surgery, Surgical complications, Classification, Organ dysfunctions

## Abstract

**Background:**

Patients undergoing emergency surgery have a high risk for surgical complications and death. The Clavien-Dindo classification has been developed and validated in elective general surgical patients, but has not been validated in emergency surgical patients. The aim of the current study was to evaluate the Clavien-Dindo classification of surgical complications in emergency surgical patients and to study preoperative factors for risk stratification that should be included into a database of surgical complications.

**Methods:**

A cohort of 444 consecutive patients having emergency general surgery during a three-month period was retrospectively analyzed. Surgical complications were classified according to the Clavien-Dindo classification. Preoperative risk factors for complications were studied using logistic regression analysis.

**Results:**

Preoperatively 37 (8.3%) patients had organ dysfunctions. Emergency surgical patients required a new definition for Grade IV complications (organ dysfunctions). Only new onset organ dysfunctions or complications that significantly contributed to worsening of pre-operative organ dysfunctions were classified as grade IV complications. Postoperative complications developed in 115 (25.9%) patients, and 14 (3.2%) patients developed grade IV complication. Charlson comorbidity index, preoperative organ dysfunction and the type of surgery predicted postoperative complications.

**Conclusions:**

The Clavien-Dindo classification of surgical complications can be used in emergency surgical patients but preoperative organ dysfunctions should be taken into account when defining postoperative grade IV complications. For risk stratification patients’ comorbidities, preoperative organ dysfunctions and the type of surgery should be taken into consideration.

## Background

The quality of care and patients’ safety has gained a lot of interest during the last decade. Morbidity and mortality rates of surgical patients may serve as one method for quality assessment [1]. Clavien and Dindo proposed a classification for postoperative complications [2] that was revised and validated later on [3]. The Clavien-Dindo classification is a modified version of the initial classification of complications published in 1992 [4]. In addition to the Clavien-Dindo classification, the accordion severity grading system for surgical complications [5] was derived from the same initial classification system [4]. In this initial classification and its later derivatives, complications are differentiated from failure to cure and sequelae. The Clavien-Dindo classification defines a complication as any deviation from normal postoperative course. The Clavien-Dindo classification has gained popularity in many fields of surgery and its use is highly recommended for reporting complications after urologic surgical procedures [6].

The Clavien-Dindo classification was developed and validated using a cohort of general elective surgical patients [2]. However, patients undergoing emergency general surgical procedures differ significantly from elective general surgical patients. Although emergency surgical patients represent a minority (11%) of all general surgical patients, they are associated with half of mortalities and nearly one third of the complications [7]. Therefore, emergency surgical patients are an important target group for quality improvement, and negative outcomes should be measured and classified in order to find more specific targets for quality improvement. The suitability of the Clavien-Dindo classification for emergency surgical patients has not been validated. Particularly, organ failures classified as grade IV complications in the Clavien-Dindo classification may be inappropriate for classifying complications of emergency surgical patients, who may already have organ failures preoperatively caused by a severe disease or injury.

The present study was conducted to validate the Clavien-Dindo classification in general emergency surgical patients and to provide guidelines and suggestions for the use of this classification in emergency surgical patients. Secondly, this study was conducted in order to develop a valid tool for prospective monitoring of morbidity and mortality with the inclusion of relevant preoperative data for risk adjustment of postoperative complications.

## Methods

Institutional review board approved the present study. All emergency general surgical procedures between 21^st^ April 2012 and 23^rd^ June 2012 were retrospectively identified from an administrative database of surgical procedures in a single academic teaching hospital. Emergency general surgical procedures included also re-operations for complications after elective surgery. A retrospective data collection included preoperative characteristics including co-morbidities for the calculation of the Charlson comorbidity index [8], functional status, the type of residence, organ dysfunctions, perioperative data, and postoperative complications. Preoperative organ dysfunctions were defined as organ specific sequential organ failure assessment (SOFA)-score of 2 or more [9] or if a patient required preoperative intermediate level care (IC) or intensive care unit (ICU) management for organ dysfunction.

A trained nurse, with the help of a senior surgeon as needed, performed a retrospective chart review after a minimum of 30 days after surgery. After initial data collection the charts of all patients with surgical complications were re-reviewed by the senior surgeon to confirm the initial classification. Mortality data was re-checked after six months after operation from the Finnish population register. In patients having more than one operation for the treatment of the same condition, only the index emergency surgery procedure was included in the analysis. Complications were classified according to the Clavien-Dindo classification. Organ dysfunctions were classified as complications if the patient developed new organ dysfunction after surgery. If a patient had organ failure before operation and needed postoperative ICU management for this, it was not considered as complication but as failure to cure. All deaths within 100 days from surgery were classified as Grade V complications irrespective of the cause of death as proposed by in the Accordion Severity Grading System [5].

Statistical analysis was performed using IBM SPSS Statistics Version 20 for Macintosh. Odds ratios for complications were calculated for preoperative risk factors and logistic regression analysis was used to assess the relevant risk factors that should be included in future data collection of postoperative complications.

## Results

During the study period 447 patients with 518 emergency general surgery procedures were identified. Three patients did not live in Finland and were discharged early postoperatively and thus did not have reliable data for assessment of post-operative complications and were excluded from the analysis. The remaining 444 patients were included in the analysis. The patients’ baseline characteristics are shown in Table 1. Intra-abdominal infections were the most frequent cause of emergency surgery as shown in Table 2. According to the operation codes obtained from the database, 59 different kinds of index operations were performed. Table 3 shows the distribution of most frequently performed procedures. Thirty-eight patients (8.6%) had to be re-operated after the index operation and 18 patients (4.1%) had more than one re-operation. Twelve patients (2.7%) had a planned re-operation at least once. 115 (25.9%) out of 444 patients developed postoperative complications. Table 4 shows the length of hospital stay and the distribution of complications according to the Clavien-Dindo classification. In surviving patients, hospital stay increased along with the increasing complication grade (p < 0.0001, Spearman rank correlation test). Fifty-seven (49.6%) patients had a complication, which had a causal relationship with the surgical procedure. Other complications included any deviation from the normal postoperative course without a direct causal effect.

**Table 1:** Characteristics of the 444 patients undergoing emergency surgery

**Characteristics**	**All patients (n = 444)**
Female sex	231 (52%)
Age, median (range)	50 (16 – 93)
Age over 80 years	40 (9%)
Function state (activity of daily living)	
Independent	420 (95%)
Partially dependent	22 (5%)
Dependent	2 (0.5%)
Living at home before hospitalization	426 (96%)
Comorbidities	214 (48%)
Charlson index > 0	136 (31%)
Charlson index, mean (range)	2.5 (1–9)*
Charlson index ≥ 2	84 (19%)
Any malignancy	50 (11%)
Metastatic solid tumour	16 (3.6%)
Preoperative organ dysfunction	37 (8.3%)
Reason for emergency surgery complication	32 (7.2%)
Surgical complication	19 (4.3%)
Other complication (including endoscopic etc.)	13 (2.9%)
Referral patient	14 (3.2%)
Previous surgery within 30 days	19 (4.3%)

The numbers represent number of patients (%) unless stated otherwise.

*Patients with Charlson index >0.

**Table 2:** Reasons for emergency general surgery in the 444 patients according to findings in surgery

**Condition**	**Number of patients (%)**
Uncomplicated intra-abdominal infection*	211 (47.5%)
Bowel obstruction	52 (11.7%)
Peritonitis	48 (10.8%)
Incarcerated hernia	34 (7.7%)
Diagnostic exploration - no findings	32 (7.2%)
Intra-abdominal abscess	15 (3.4%)
Extra abdominal infection	17 (3.8%)
Pancreatitis**	11 (2.5%)
Trauma	9 (2.0%)
Mesenteric ischemia	5 (1.1%)
Hemorrhage	4 (0.9%)
Hemorrhoids	3 (0.7%)
Biliary colic	3 (0.7%)

*Includes uncomplicated appendicitis and cholecystitis and other uncomplicated infections.

**Includes cholecystectomy for biliary pancreatitis, surgery for abdominal compartment syndrome and surgery for infected pancreatic necrosis.

**Table 3:** Emergency surgery operations in 444 patients

**Procedure**	**Number of patients (%)**
Laparoscopic appendectomy	145 (32.7%)
Laparoscopic cholecystectomy	55 (12.4%)
Any colon resection	43 (9.7%)
Open appendectomy	37 (8.3%)
Open cholecystectomy	14 (3.2%)
Incision and drainage of perianal abscess	14 (3.2%)
Repair of umbilical hernia	14 (3.2%)
Small bowel resection	13 (2.9%)
Repair of inguinal hernia	12 (2.7%)
Explorative laparotomy	12 (2.7%)
Open adhesiolysis	10 (2.2%)
Open repair of peptic ulcer perforation	9 (2.0%)
All other procedures	66 (14.9%)

**Table 4:** Most severe postoperative complication and hospital stay in 444 patients after emergency surgery

**Complication grade**	**Number of patients (%)**	**Hospital stay**^**†**^**, days Median (IQR)**
No complication	329 (74.1%)	2 (1 – 4)
I	7 (1.6%)	2 (1 – 6.5)
II	32 (7.2%)	4 (1.5 – 7.5)
III	32 (7.2%)	9 (5.5 – 24.5)
IIIa	14 (3.2%)	9.5 (6 – 30)
IIIb	18 (4.1%)	8.5 (3 – 20)
IV	14 (3.2%)	38.5 (14 – 78)
IVa	1 (0.2%)	53
IVb	13 (2.9%)*	35 (14 – 78)
V	30 (6.8%)	5.5 (2 – 12)

*Four patients had preoperative organ dysfunction.

^†^Hospital stay after emergency surgery, IQR inter quartile range.

The cause of death was cancer in 10 (33%) out of 30 patients classified as grade V complication, with a median time to death of 23 days (range 2 to 66 days) post surgery. The second most common cause of death was multi-organ failure in 8 patients, with median time to death of one day (range 0 to 11 days) post surgery. Nine patients died after 30 days but before 100 days after surgery. During the 6 months after surgery, 33 patients died, 3 of these were not included in grade V complications defined by death within 100 days after surgery, because the time of death was 115, 124 and 168 days after surgery in these patients. These patients had complications classified as grade IIIb, IIIa and IVb, respectively. One of them died because of incurable cancer, but in two patients the causes of deaths were associated with surgical complications. These included cholangitis after biliary stenting for postoperative biliary fistula and intra-abdominal abscess after necrotizing pancreatitis, respectively.

Table 5 shows that several factors were significantly associated with postoperative complications (Table 5). Using logistic regression analysis it was found that the Charlson co-morbidity index, preoperative organ dysfunctions, and laparotomy were independently predicting post-operative complications (Table 6). Surgery due to surgical complication was also an independent risk factor for post-operative grade III – V complications (Table 6).

**Table 5:** Risk factors for post-operative complications (all grades of complications) in univariable analysis

**Risk factor**	**Odds ratio**	**95% CI**
Age over 55 years	3.8	2.4 – 6.0
Charlson index > 1	4.7	3.0 – 7.4
Charlson index ≥3	6.6	3.5 – 12.7
Female sex	0.6	0.39 – 0.92
Preoperative organ dysfunction	6.4	3.1 – 13.1
Laparotomy*	6.3	3.9 – 10.0
Surgery because of surgical complication	3.4	1.3 – 8.6
Surgery because of other iatrogenic complication	1.3	0.39 – 4.2**
Time of surgery during regular working hours	0.83	0.53 – 1.3**
Resident as primary surgeon	0.4	0.26 – 0.62
Functional state: partially or totally dependent	2.6	1.1 – 5.9
Living at home before hospitalization	0.53	0.20 – 1.4**

*excluding open appendectomy.

**not significant.

CI – confidence interval.

**Table 6:** Independent predictors of all postoperative complications and grade III-IV complications in multivariable logistic regression analysis*

**Risk factors**	**Odds ratio**	**95% confidence interval**
**All complications**		
Charlson index	1.34**	1.1 – 1.6
Preoperative organ dysfunction	4.4	2.0 – 9.4
Type of surgery: laparotomy	4.1	2.5 – 6.8
**Grade III – V complications**		
Charlson index	1.3**	1.1 – 1.5
Preoperative organ dysfunction	5.0	2.2 – 11.2
Type of surgery: laparotomy	8.4	4.3 – 16.3
Surgery because of surgical complication	3.2	1.002 – 10.2

*Stepwise forward analysis was done including age, sex, Charlson index, preoperative organ dysfunction, type of surgery, surgery done for surgical complication, surgery done for other iatrogenic complication, time of surgery, primary surgeon, functional state and residency into the model.

**Odds ratio for increase of index by 1.

In patients who had emergency laparotomy (n = 154), the overall the rate of complications was 76/154 (49.3%) and the mortality rate was 28/154 (18.2%). In contrast, in patients having laparoscopic surgery or other minor surgery the respective overall complication rate was 39/290 (13.4%) and mortality rate 2/290 (0.7%), p < 0.001 for both. In addition, the increasing number of preoperative organ dysfunctions (0, 1, 2 and 3 organ dysfunctions) increased the overall complication rate (22.4%, 52.2%, 83.3% and 100%, respectively) and the 100-day mortality rate (4.2%, 26.1%, 50.0% and 50.0%, respectively), p < 0.001 for both.

## Discussion

The Clavien-Dindo classification defines surgical complication as any deviation from the ideal postoperative course that is not inherent in the procedure and does not comprise a failure to cure. Condition that remains unchanged after surgery is not a complication but rather a failure to cure. Because this classification was developed and validated with elective general surgical patients, little guidance was given how this classification should be used in patients undergoing emergency surgery. In clinical scenarios provided by Clavien et al. [3] only two scenarios with acute condition were presented: In these two scenarios the patient with acute severe cholecystitis dies either during the operation or preoperatively during the preparation of the surgery. The guideline was given to classify the both these deaths as a grade V complication.

This study shows, that the Clavien-Dindo classification can be applied also for patients undergoing emergency surgery. However, the prerequisite for this is that patients’ preoperative organ dysfunction status is assessed. Preferably the presence of preoperative organ dysfunctions should be included in the final complication grade as an addendum indicating that the patient had organ dysfunction before surgery. Because peritonitis [10] or severe bleeding due to trauma [11] may cause organ dysfunctions, all postoperative organ dysfunctions should not be classified as surgical complications. In this study postoperative organ dysfunctions were not classified as complications if it they were already present preoperatively i.e. the patient’s condition remained unchanged in terms of organ dysfunctions. However, if a patient developed other postoperative complications that probably caused prolonged organ dysfunctions or worsening of organ dysfunctions, the complication was classified as grade IV. Also any progression of organ failure to death was classified as grade V complication. Because surgical trauma can cause postoperative organ dysfunctions as shown in patients operated for infected pancreatic necrosis [12], any new organ failure occurring after surgery is a deviation from ideal postoperative course and should be classified as a complication according to the Clavien-Dindo classification. Although in many patients with severe trauma or peritonitis these complications may be inevitable, the classification of complications should be as objective as possible leaving minimal room for subjectivity.

In the Clavien-Dindo classification, transferred patients from other institutions are identified with an addendum (referred patient). According to the guidelines provided [3] every patient operated for postoperative complications are classified as having a surgical complication. In this study patients operated on for postoperative complications after elective surgery were classified according to same principles as other emergency surgery patients. By using this approach, complications after re-operations can be classified and the quality of re-operations monitored.

In the Clavien-Dindo classification organ dysfunctions are defined as any life threatening complication requiring IC/ICU treatment. The time frame from hospital admission to operating room can be very short in emergency surgical patients, so that they are very seldom admitted to IC/ICU unit before surgery. Therefore, other definitions for preoperative organ dysfunctions should be established. The Accordion severity grading system provides alternative solution for the definition of organ failure [5]. Indeed, there are several possible ways to define organ dysfunction using multiple organ dysfunction scores [9,13] or their modifications [14].

In this study we defined preoperative organ dysfunction as SOFA-score 2 or more in any organ system [9], which is comparable with the recent definition used in defining organ dysfunctions in acute pancreatitis [14]. The reason for the use of SOFA-score is that it is routinely used in the ICU in our hospital and this makes comparison of preoperative and postoperative organ dysfunctions possible. However, there are some limitations with the use of the SOFA-score in emergency setting. The RIFLE criteria are more sensitive for acute renal failure and could serve as an alternative for preoperative identification of renal dysfunction [15]. The definition of postoperative organ dysfunction was identical with the Clavien-Dindo classification, but postoperative organ dysfunction was not considered as a complication if present preoperatively. A few clinical scenarios are provided for clarification how to classify patients’ complications when organ dysfunctions complicate the clinical course (Table 7).

**Table 7:** Eight clinical scenarios of emergency surgical procedures and classification of complications

**Scenario**	**Description and classification**
1	A patient with generalized peritonitis was admitted to the hospital. The patient was in septic shock and large volume fluid resuscitation and vasopressor medication were administered in the emergency department. Laparotomy was performed and a perforated diverticulitis was managed with the Hartmann’s procedure. Postoperatively the patient was in the ICU for 6 days, recovered from septic shock and was discharged from hospital 2 weeks later.
	Classification: (preoperative organ dysfunction), no complication
2	The same patient as in scenario 1, but resection and primary anastomosis was performed. Postoperatively the patient was admitted into the ICU. After 7 days patient had not recovered, and increasing doses of norepinephrine were needed. Computed tomography on seventh postoperative day showed a large (10 cm) pelvic abscess. The patient was re-operated and anastomotic leakage was managed by resection of the anastomosis and end-colostomy. After the second operation the patient recovers and is transferred to regular ward after 13 days and discharged from hospital 4 weeks later.
	Classification: (preoperative organ dysfunction), grade IVb complication
3	An elective laparoscopic sigmoid resection due to diverticular disease was performed in another hospital in a 62-year old man. The patient was discharged in good condition on the second postoperative day according to enhanced recovery after surgery program. On the sixth postoperative day the patient develops severe abdominal pain and presents to our hospital with signs of peritonitis. While waiting for a CT scan his blood pressure drops and despite fluid resuscitation vasopressors are needed to maintain adequate blood pressure. Free air was found in the CT, and anastomotic dehiscence was found in emergency laparotomy requiring a Hartmann’s procedure. Postoperatively the patient recovered from shock and stayed in the ICU for two days, and was discharged from hospital 10 days later.
	Classification: (preoperative organ dysfunction), no complication Note: Surgical unit, that had done elective surgery, should classify this patient as grade IV complication.
4	A 73-year old woman was admitted to the hospital with a sudden onset of severe abdominal pain. She had signs of peritonitis and free air on CT-scan, but no organ dysfunctions. Patient aspirated during intubation in the operating room. Emergency laparotomy was performed and a perforated duodenal ulcer was sutured with an omental patch. Postoperatively the patient developed signs of MODS, required mechanical ventilation, vasopressor support and was oliguric. She was transferred to the ICU where bilateral pneumonia was diagnosed later. After ten days of intensive care the patient was transferred to the regular ward, and she was discharged from hospital three weeks later.
	Classification: (no preoperative organ dysfunction), grade IVb complication
5	A 23-year old hockey player was admitted to hospital due to blunt grade IV splenic trauma. He was haemodynamically stable, but the haemoglobin level was 70g/l and in the CT-scan there was a lot of intra-abdominal blood. Emergency laparotomy and splenectomy were performed. However, during the surgery there were technical difficulties to achieve haemostasis and the operation lasted for three hours. Due to bleeding the patient received 12 units of packed red blood cells and 8 litres of crystalloids during the first 24 hours. Postoperatively the patient was admitted to the ICU, required vasopressors during the first day and was mechanically ventilated for 2 days. The patient recovered and was discharged from hospital 6 days later.
	Classification: (no preoperative organ dysfunction), grade IVb complication
6	The same patient in scenario 5, but the operation was performed without problems in 40 minutes. The patient was extubated after surgery and transferred to the regular ward postoperatively. The patient got only 4 units of packed red blood cells and 4 litres of crystalloids. The postoperative course was uneventful.
	Classification:(no preoperative organ dysfunction), no complication
7	A 31-year old man with abdominal gunshot wound was admitted to the hospital. He was in haemorrhagic shock and required endotracheal intubation. The patient was transferred directly to the operating room, and emergency laparotomy was performed. Multiple bowel injuries and bleeding from mesenteric and left iliac vessels were found. Damage control laparotomy with good haemostasis was performed and the abdomen was left open at the end of the operation. The patient received 20 units of packed red blood cells and 12 litres of crystalloids. Postoperatively the patient was transferred to the ICU, mechanically ventilated and recovered from shock during the first postoperative day. Thirty-five hours later a planned re-operation was performed with restoration of bowel continuity and closure of the abdomen. The patient was extubated on the fifth day and transferred to the regular ward. The patient recovered and was discharged 12 days later.
	Classification: (preoperative organ dysfunction), no complication
8	A 42-year old man with drug addiction was admitted to the hospital because of multiple abdominal stab wounds. He was haemodynamically stable but had signs of peritonitis. Emergency laparotomy was performed and two traumatic perforations in the transverse colon and one perforation in the ileum were identified and sutured. Postoperatively the patient was treated in the regular ward. He had constant pain requiring repeated doses of opioids. On the third postoperative day the patient developed shock, was transferred to the ICU by the medical emergency team and a CT-scan was performed. There was a considerable amount of peritoneal fluid, but no free air. An explorative laparotomy was performed and a missed perforation in the jejunum was found and sutured. Postoperatively the patient recovered uneventfully.
	Classification: (no preoperative organ dysfunction), grade IVa complication

Postoperative death should always be classified as complication, because it obviously is a deviation from the ideal post-operative course. Although in some patients undergoing emergency surgery the death may not be preventable [16], we encourage classifying also these deaths as complications in order to avoid subjective interpretations. As presented here the time frame for monitoring postoperative deaths should be longer than 30 days. By limiting the follow-up from 100 days to 30 days would have reduced mortality from 6.8% to 4.7%. As proposed by Strasberg et al. [4] a 100-day mortality could be a good time frame for monitoring postoperative deaths. Nevertheless, it is important to report the follow-up time when reporting complications.

Some patients are more prone to develop complications than other patients. The type of surgery and the condition of the patient have a major impact on the risk of developing complications. Therefore it is important to register also the type of surgical procedure and patient’s preoperative condition to the database of surgical complications. Based on the results we recommend, that patients’ preoperative organ dysfunctions should be monitored and included in registration of complications. The Charlson co-morbidity index could serve as a measure of co-morbidities. Emergency surgery due to surgical complication increased significantly the risk of grade III –V complications, and therefore it would also be another important factor to be registered.

Another finding in this study was that the majority of emergency general surgery patients have minor operations, mainly laparoscopic surgery with low rate of complications. On the other hand, nearly half of the patients having emergency laparotomy developed complications and nearly one fifth of the patients died. These differences may be explained by selection process where laparoscopic surgery was used more often for minor operations whereas laparotomy was used more often in complex procedures and in high-risk patients. Nevertheless, patients having emergency laparotomy need to be closely monitored for surgical complications and every effort must be taken to improve the quality of care in these patients.

## Conclusions

The Clavien-Dindo classification of surgical complications can be used in emergency surgery patients. Organ dysfunctions prior to surgery should be taken into account in order to define when postoperative organ dysfunction is a complication. For risk adjustment, patients’ co-morbidities, preoperative organ dysfunctions and the type of surgery should be included in the database of surgical complications.

## Abbreviations

IC: Intermediate level care; ICU: Intensive care unit; SOFA: Sequential organ failure assessment.

## Competing interests

The authors declare that they have no competing interests.

## Authors’ contributions

PM designed the study, participated in acquisition of the data, did statistical analysis, and drafted the manuscript. AL participated in designing of the study and interpretation of the data and revised the manuscript. Both authors read and approved the final manuscript.
